# Who Is the Rightful Owner? Young Children’s Ownership Judgments in Different Transfer Contexts

**DOI:** 10.3389/fpsyg.2018.01314

**Published:** 2018-07-31

**Authors:** Zhanxing Li, Minli Qi, Jing Yu, Liqi Zhu

**Affiliations:** ^1^CAS Key Laboratory of Behavioral Science, Institute of Psychology, Chinese Academy Sciences, Beijing, China; ^2^Institute of Social Psychology, School of Humanities and Social Sciences, Xi’an Jiaotong University, Xi’an, China; ^3^National Institute of Child Health and Human Development, Bethesda, MD, United States

**Keywords:** ownership reasoning, ownership transfers, first possessor bias, giving and stealing, losing and abandoning

## Abstract

This study aimed to examine whether Chinese preschoolers understand that ownership can be transferred in different contexts. The study participants were 3- to 5-year-old Chinese children (*n* = 96) and adults (*n* = 34). With four scenarios that contained different transfer types (giving, stealing, losing, and abandoning), participants were asked four questions about ownership. The results indicated that preschoolers’ ability to distinguish legitimate ownership transfers from illegitimate ownership transfers improved with age. Three-year-olds understood that ownership cannot be transferred in a stealing context, but the appropriate understanding of ownership was not attained until 4 years old in a giving context and 5 years old in losing and abandoning contexts, which is similar to the adults’ performance. In addition to the first possessor bias (a tendency to judge the first possessor as the owner) found in previous studies, 3-year-olds also displayed a loan bias (a tendency to believe everything that is transferred should be returned) in the study. The findings suggest that the developmental trajectories of preschoolers’ understanding of ownership transfers varied across different contexts, which may relate to children’s ability to consider the role of intent in determining ownership and parents’ disciplinary behavior. Both cross-cultural similarities and differences are discussed.

## Introduction

Ownership plays an important role in our daily lives. People with no awareness of ownership could take others’ objects without permission and incur social conflict (Ross and Conant, 1992; Friedman and Ross, 2011; Ross, 2013). Property rights have been found in many human cultures (Brown, 1991; Friedman and Ross, 2011). To maintain a normal social order, many countries have established property laws (Dari-Mattiacci and Guerriero, 2015), by which the legitimate rights of ownership are defended and the illegitimate seizure of ownership is punished. Compliance with these property laws helps people avoid many unnecessary troubles in daily life.

Researchers have argued that even adults and young children who are not familiar with property laws have an intuitive understanding of ownership and make ownership judgments based on property principles in law, such as first possession and labor (Locke, 1690/1978; Epstein, 1978). Adults and preschoolers tend to perceive that an unowned object belongs to the person who first possessed it (Friedman and Neary, 2008; Friedman et al., 2013; Nancekivell et al., 2013), and acknowledge that ownership can be transferred via labor to a modifier or a creator (Kanngiesser et al., 2010; Kanngiesser and Hood, 2014; Levene et al., 2015). Two-year-olds can use verbal testimony to identify the owner, attributing a toy to the person who claims ownership of it (Blake and Harris, 2011; Blake et al., 2012). Three-year-olds can reason that a toy belongs to the person who permits others to play it (Neary et al., 2009), and by age six, children can use this principle to judge ownership of intellectual property (Shaw et al., 2012). These studies demonstrate that even young preschoolers have grasped the basic ability to reason about ownership according to different rules and cues in contexts.

However, younger preschoolers may still have difficulty in inferring ownership in some contexts, especially when the object has been transferred. Noles and Gelman (2014) found that 4-year-olds could judge the gift recipient as the owner of the present regardless of whether he/she likes it or not. Moreover, Kim and Kalish (2009) found that even 4–5-year-old children understand that ownership rights can be transferred. They were able to track owners’ rights across transfers, such as gift-giving, and judged that current owners have control of objects over non-owners. By contrast, Friedman and Neary (2008) found that 3- and 4-year-olds would judge that the gift giver was still the owner of the present if the present was unwrapped and the first possessor played with it. If a toy was wrapped and given as a birthday present, young children were more likely to judge that the recipient was the owner. Blake and Harris (2009) investigated 2- to 5-year-old preschoolers’ understanding of ownership transfers in giving and stealing contexts. Those authors found that although most 5-year-olds judged that ownership can be transferred in the giving context and cannot be transferred in the stealing context, 2- and 3-year-olds often denied the change in ownership in the giving context. Younger preschoolers seem to have a *first possessor bias* in ownership judgments in transfer contexts of giving and stealing (Friedman and Neary, 2008; Blake and Harris, 2009; Friedman, 2009; Kim and Kalish, 2009) and cannot discriminate legitimate transfers from illegitimate transfers as older children can (Blake and Harris, 2009).

Two other common transfers, losing and abandoning, have been somewhat neglected in previous studies. Similar to transfers in stealing and giving contexts, transfers in losing and abandoning contexts also correspond to illegitimate and legitimate acquisition of ownership (Ma, 2003; Simeone, 2009). Conventionally, a man who has lost his property has not given up ownership of it, but when someone abandons an object, it typically means that he does not want it and that anyone who finds it first can be its owner (Ma, 2003; Simeone, 2009). To our knowledge, only one study has involved young children’s ownership judgments about lost properties (Cram and Ng, 1989), and it showed that 5- to 12-year-old children’s endorsement of legitimate attributes increases with age. While older children endorsed contractual attributes arguing that lost properties should be given back to its original owner, many younger children endorsed physical attributes arguing that finders could keep lost properties because they found it. However, that study did not directly examine children’s understanding of ownership transfer.

Whether in the giving and stealing contexts or in the losing and abandoning contexts, it is the original owner’s intent that determines ownership status. Though children as young as 18-month-olds can detect the intent of an actor by watching his/her behavioral attempts (Meltzoff, 1995), young children often mix actors’ intents with their desires and outcomes (Feinfield et al., 1999). In addition, young children cannot fully understand the role of intent in guiding causal inference. For example, when asked to make moral judgments, preschoolers often give more weight to outcome than to intent (Piaget, 1932; Cushman et al., 2013). Younger preschoolers are more likely to judge that a boy who accidentally breaks a mirror should be punished than older children. As they grow up, children make moral judgments increasingly more on the basis of actors’ intent, as opposed to the outcome of actors’ actions (Cushman et al., 2013). Actors’ intent in the context is crucial to both moral judgments and ownership judgments, and preschoolers may have difficulty in understanding intent in reasoning about ownership, as in moral judgments.

In addition, the development of ownership reasoning may vary across different contexts. Giving behavior is often encouraged as a prosocial behavior, whereas stealing behavior is often punished as an immoral behavior in children’s early life. Young children may develop the concept of ownership earlier in these two contexts than do children in other contexts. Parents respond more strongly to children’s antisocial behaviors (Grusec and Goodnow, 1994), but they may rarely intervene when their children find property in lost and abandoned situation; thus, children’s understanding of ownership transfer in these two contexts may develop later. Educational practices from parents may influence children’s understanding of ownership in illegal transfers and legal transfers. It is worthwhile to investigate children’s ownership judgments in different contexts because they may not develop synchronously.

Notably, most previous research concerning children’s ownership judgments has been conducted in Western countries. A few studies with Asian children showed both cross-cultural similarities and differences in children’s ownership understanding (Kanngiesser et al., 2014, 2015; Rochat et al., 2014; Yang et al., 2014). Yang et al. (2014) found that both American and Chinese 5-year-old children thought that plagiarizing another person’s ideas was illegitimate, since the ideas belong to the people who first have them. Kanngiesser et al. (2014, 2015) revealed that British 3–4-year-olds judge that the products belong to the creators when watching videos of conflicts between the original owner and creators whose labor contributed to producing the materials, whereas young Chinese and Japanese children did not show a preference for the creators. Rochat et al. (2014) found that the development of children’s ownership reasoning based on first possession and creation was similar across American and Chinese cultures, but Chinese children tended to split the object into equal halves whenever possible, even if this does not accord with ownership principles. We expect both similarities and differences in conditions, comparable to those found in previous studies with Western children.

In sum, we aimed to investigate Chinese preschoolers’ ownership judgments across four transfer conditions (i.e., giving, stealing, losing, and abandoning) in this study. To our knowledge, this is the first study to explore Chinese children’s understanding of ownership transfers and the first study to include a context for abandoned property. We expected the development of children’s understanding of ownership to differ among the four transfer contexts. Specifically, for younger preschoolers, because of their limited capacity to understand the role of intent, they may have difficulty in ownership judgments. Moreover, because illegal acts are often given more attention in children’s lives, it may be easier for children to reason about ownership in illegal transfer contexts than in legal transfer contexts.

We recruited 3- to 5-year-old Chinese preschoolers as the study group and a group of adults as comparison. We presented participants with scenarios involving different transfers and asked them questions concerning ownership status at the end of the scenarios. We also asked participants to justify their answers so that we could analyze any biases they may hold. In previous studies, in addition to the aforementioned *first possessor bias*, two other types of biases were also revealed—the *current possessor bias* and the *loan bias* (Beggan and Brown, 1994; Blake and Harris, 2009; Friedman, 2009). The current possessor bias refers to the tendency to judge the current possessor of the object to be the owner (Beggan and Brown, 1994; Blake and Harris, 2009). The loan bias refers to the tendency to believe that everything that is transferred should be returned (Blake and Harris, 2009). The loan bias is relevant only when a subject is asked whether the property should be returned. Based on the findings in previous studies (Friedman and Neary, 2008; Blake and Harris, 2009; Friedman, 2009; Kim and Kalish, 2009), we hypothesized younger children may have the first possessor bias in ownership judgments in general. Given that Chinese cultures discourage people from keeping property that they did not earn with effort, the loan bias may be found in our study.

## Materials and Methods

### Participants

A total of ninety-six 3- to 5-year-olds from three classrooms participated in this study. They were recruited from two ordinary kindergartens in Shanghai, China. For the 3-year-old group, 32 children were tested initially, but two of them did not complete the task. Thus, the final sample included 3-year-olds (*n* = 30, *M* = 3.56, *SD* = 0.30, age range = 3.10–3.97, 14 boys), 4-year-olds (*n* = 32, *M* = 4.53, *SD* = 0.29, age range = 4.01–4.99, 16 boys) and 5-year-olds (*n* = 32, *M* = 5.65, *SD* = 0.26, age range = 5.00–5.94, 14 boys). All the preschoolers were of Han ethnicity and were mandarin speakers. Approximately 63% of the children’s primary caregivers had received a high school education or higher, and 49% had received a university education or higher. Most parents self-reported having a middle level income. Caregivers signed an informed consent form before their children participated in the study. The study was conducted with the approval of the institutional Scientific Research Ethics Committee of our university. In addition, thirty-four college students (*M* = 24.56, *SD* = 1.46, age range = 22.08–28.16, 17 males) were recruited via the popular network communication tool Tencent Instant Messenger from a university in Shanghai. The students were asked to sign an informed consent form before participating in the experiment.

### Materials

We used four scenarios that depicted four transfer events (i.e., giving, stealing, losing, and abandoning) as materials. For the scenarios of giving and stealing, we used two scenarios similar to those used in the study of Blake and Harris (2009), but substituted the protagonists with Chinese names (e.g., Qiangqiang, Xiaole). The scenarios were adapted to reflect the other scenarios of losing and abandoning (see the **Appendix**). We conducted a pilot study with a different group of 3- to 5-year-olds and found that the subjects had no preference for the protagonists’ names.

To make it easier for preschoolers to understand the scenarios, we matched the scenarios with four sets of black and white line drawings. Each set of drawings was depicted on a sheet of paper with a size of 195 mm × 271 mm and was divided into four settings. We designed two sets of materials (i.e., scenarios and pictures) separately to match the subjects’ genders. Protagonists were differentiated by their names and clothes. For the toys that were used as target objects in the scenarios, we selected eight toys commonly seen in Chinese families and kindergartens: for boys, we selected a toy car, a wooden horse, a mini-telescope, and a football; for girls, we selected a Mickey Mouse, a toy bear, a Barbie doll, and a paper fan. All children disclosed that they were familiar with these toys before beginning the study.

### Procedure

The study was carried out by an experimenter, a female graduate psychology student who was blind to the experimental hypothesis. Preschoolers were invited into a quiet room in the kindergarten class, and they sat on one side of a table facing the experimenter. First, the experimenter played with the child for 1 or 2 min to make him/her comfortable. Then, the experimenter said, “I’m going to tell you some stories and ask you some questions about the stories, OK?” While the experimenter narrated the scenarios, she pointed to the drawings and the protagonists to keep the child on track. The following is an example of a scenario and drawing in the giving condition:


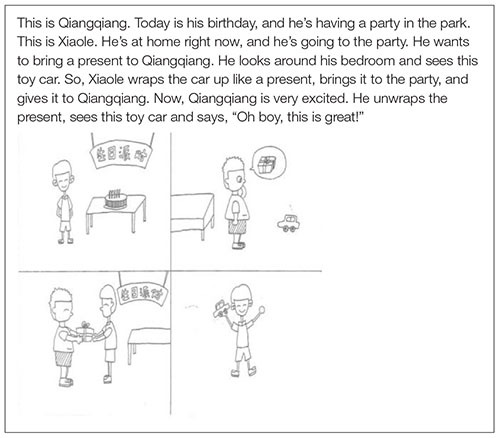


After hearing the scenarios, children were first asked a memory recall question: “At the beginning of the story, whose toy car was it?” The scenarios were told again if children failed to give a correct answer to the memory recall question until they remembered the original owner in the scenario. They were then asked four test questions: (1) At the end of the story, is the toy car still Xiaole’s, or is it Qiangqiang’s? (2) Who can take the toy car home now, Xiaole or Qiangqiang? (3) Qiangqiang is holding the toy car at the end of the story. Does he need to give it back to Xiaole? (4) Why?

In the stealing condition, children were told about a scenario in which a protagonist stole another protagonist’s toy when he/she was absent. In the losing condition, they were told about a scenario in which a protagonist forgot his/her toy on a bus and another protagonist picked up the toy. In the abandoning condition, they were given a scenario in which a protagonist threw his/her toy away next to a trash bin, and another protagonist picked up the toy. The same procedure as used in the giving scenario was applied to these three scenarios. Because of the small sample size for each age group, the stories were partially counterbalanced in four different orders: (1) giving, stealing, losing, abandoning; (2) stealing, losing, abandoning, giving; (3) losing, abandoning, giving, stealing; and (4) abandoning, giving, stealing, losing. Such a way of presenting scenarios can ensure that each story was told in each order eight times in each age group. Participants were randomly assigned to one of the four orders. The names of two protagonists in each scenario were also counterbalanced. Adults were provided with a piece of paper with four scenarios without pictures and were asked to write down their answers on the paper. They were asked to read the scenarios and make their decisions independently. Some of them answered that the toy could be owned or played with by both protagonists together at first. In that case, they were asked to choose only one protagonist in the scenario. Participants were given either an eraser (for preschoolers) or a pen (for college students) as compensation.

### Scoring and Coding

For the first three questions, we scored each answer as 1 if it supported the first possessor in the stealing and losing conditions and supported the current possessor in the giving and abandoning conditions. If their choices were reversed, the answers were scored as 0. We summed them to compute a total score for each condition that range from 0 to 3 as an index of children’s overall ownership judgments.

For Question (4), we found that children’s explanations varied in different conditions, but many of them referred to the original or current state of toys, the actors’ mental activities, or the legitimacy of the transfer behavior. Thus, we coded their explanations into seven mutually exclusive categories: (1) *Original state of the object*. The justification referred to the original state of the toy, such as “It was originally taken here by him/her.” (2) *Current state of the object*. The justification referred to the current state of the toy, such as “It was given to someone now.” (3) *The original possessor’s mind*. The justification referred to the original possessor’s intention or desire, such as “He/she just forgot about it” and “He/she did not want it.” (4) *The current possessor’s mind*. The justification referred to the current possessor’s intention or desire, such as “He/she likes it.” (5) *The legitimacy of the behavior.* Subjects thought the act accorded with or violated rules, such as “It’s not right to take others’ things without permission.” (6) *The property of the object*. Subjects justified the answer by pointing the nature of the toy, such as “It’s a present.” (7) *Invalid response*. These responses were not informative, such as simply saying “It’s a game.” Two subjects did not give any justification in the study, and we included them in the category of *Invalid response* because they did not provide any useful information. This classification system covered all the reasons provided (see **Table 2** for more examples for each category).

The experimenter and a psychology-majored student, both of whom were blind to the experimental hypothesis, served as two independent coders. They were well-trained on the coding scheme. Several disagreements were resolved by discussion. The interrater reliability for each transfer condition ranged from 0.90 to 0.99.

## Results

### Descriptive Analysis

**FIGURE 1:**
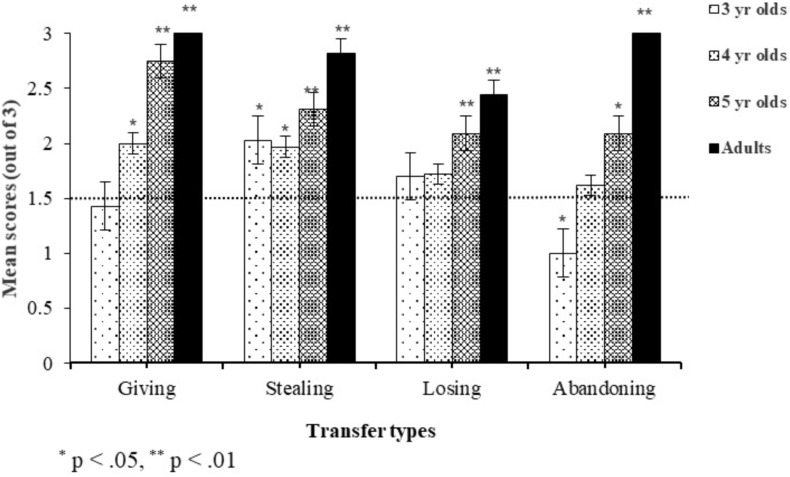
Subjects’ scores of ownership judgment in each condition.

Statistical analyses were conducted in SPSS 22.0 for Windows. **Figure 1** shows the mean scores of ownership judgments by age and transfer conditions. In general, participants’ performance in the four scenarios improved with age, but patterns of reasoning varied among different conditions. For the three child groups, they performed worst in the abandoning condition (3-year-olds: *M* = 1.00, *SD* = 0.98; 4-year-olds: *M* = 1.62, *SD* = 1.29; 5-year-olds: *M* = 2.09, *SD* = 1.25). For the adult group, however, there is a ceiling effect except for the losing condition (*M* = 2.44, *SD* = 0.93). We compared boys’ and girls’ scores in each condition but did not find any gender differences. Therefore, data from boys and girls were collapsed for the remaining analyses, which focused primarily on age and transfer condition effects.

### Scores of Ownership Judgments in Different Transfer Conditions

To examine the developmental trends of subjects’ ownership judgments in each condition, we conducted a mixed-design analysis of variance (ANOVA) with age as the between-subjects variable and transfer conditions as the within-subjects variable. The results revealed that the main effect of age was significant, *F*(3,124) = 48.75, *p* < 0.001, $\eta_{p}^{2}$ = 0.54. *Post hoc* comparisons showed significant differences across the four age groups (*p*s < 0.001), except between 3-year-olds and 4-year-olds (*p* = 0.088). The main effect of transfer conditions reached significance, *F*(3,372) = 4.77, *p* = 0.010, $\eta_{p}^{2}$ = 0.037. *Post hoc* comparisons using Bonferroni correction demonstrated that subjects’ performance was significantly better in the giving condition than that in the abandoning condition (*p* = 0.001), and that subjects’ performance was significantly better in the stealing condition than in the losing condition (*p* = 0.007).

A significant interaction between age and transfer conditions was also found, *F*(9,372) = 2.54, *p* = 0.022, $\eta_{p}^{2}$ = 0.058. A simple effect analysis showed that 3-year-olds performed significantly better in the stealing condition (*M* = 2.03, *SD* = 1.19) than in the abandoning condition, *p* = 0.006, but not significantly better than in the giving condition (*M* = 1.43, *SD* = 1.25), *p* = 0.076, and in the losing condition (*M* = 1.70, *SD* = 1.15), *p* = 0.892. Five-year-olds performed significantly better in the giving condition (*M* = 2.75, *SD* = 0.57) than in the abandoning condition *p* = 0.005, but not significantly better than in the stealing condition (*M* = 2.31, *SD* = 0.90), *p* = 0.311, and in the losing condition (*M* = 2.09, *SD* = 1.12), *p* = 0.062. Adults’ mean score in the losing condition was less than that in the other three conditions (giving: *M* = 3.00, *SD* = 0.00; stealing: *M* = 2.82, *SD* = 0.39; abandoning: *M* = 3.00, *SD* = 0.00), but these differences did not reach significance, *p*s > 0.10. There were no significant differences across the four conditions for 4-year-olds either, *p*s > 0.10.

Significant developmental differences were observed in all four conditions. In the giving condition, except for the difference between 5-year-olds and adults (*p* = 0.771), each age group performed significantly better than the relatively younger age groups (adults vs. 3- and 4-year-olds: *p*s < 0.001; 5-year-olds vs. 3-year-olds: *p* < 0.001; 5-year-olds vs. 4-year-olds: *p* = 0.002; 4-year-olds vs. 3-year-olds: *p* = 0.044). In the stealing condition, adults performed significantly better than 3-year-olds (*p* = 0.007) and 4-year-olds (*p* = 0.002), but no significant differences were found across the three preschooler groups (*p*s > 0.05). In the losing condition, adults performed significantly better than 3-year-olds (*p* = 0.048), and marginally significantly better than 4-year-olds (*p* = 0.051), but no significant differences were found across the three preschooler groups (*p*s > 0.05) or between adults and 5-year-olds (*p* = 0.741). In the abandoning condition, adults performed significantly better than all the preschooler groups (*p*s ≤ 0.001 for 3- and 4-year-olds; *p* = 0.003 for 5-year-olds). Moreover, 5-year-olds performed significantly better than 3-year olds (*p* < 0.001). No other significant differences were found.

We used a one sample *t*-test to examine whether participants’ scores in each condition were significantly above the chance level (score = 1.5). We found that adults (*p*s < 0.001) and 5-year-olds (*p*s < 0.05) performed significantly better than chance in all four conditions. In contrast, 3-year-olds’ performance was at the level of chance in the giving (*p* = 0.772) and losing (*p* = 0.348) conditions. Moreover, their performance was significantly below the level of chance in the abandoning condition (*p* = 0.009). That is, 3-year-olds showed an obvious tendency to select the first possessor as owner. Four-year-olds performed at the level of chance in the losing (*p* = 0.310) and abandoning (*p* = 0.587) conditions.

Given that a within-subjects design might be taxing for 3-year-olds, we examined their performance when each scenario was told as the first story. A one-sample *t*-test showed that the results were nearly identical, except that their ownership judgments were no longer significantly below the level of chance in the abandoning condition. Therefore, the taxing effect was trivial. In sum, the results suggested that 3-year-olds already understood that ownership cannot be transferred in the stealing condition, but such understanding was not attained in the giving condition until 4 years old and not attained in the losing and abandoning conditions until 5 years old.

### Biases in Ownership Judgments

**FIGURE 2:**
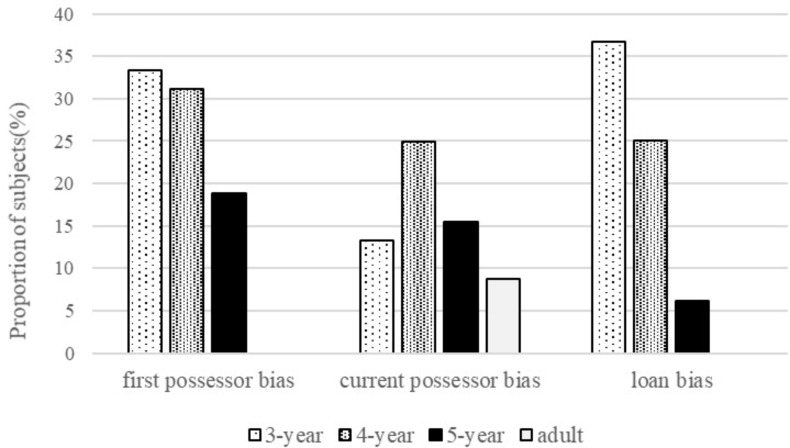
Proportions of subjects using different bias to reason ownership.

To identify the simple bias that children might use to judge ownership, we use the same method as Blake and Harris (2009)’s study, which followed a binomial distribution. Subjects’ responses were classified into three types. Those who supported the original possessor in nine of twelve questions were classified as having the *first possessor bias* (this corresponds to a cumulative binomial probability of 0.054). Those who supported the current possessor in nine of twelve questions were classified as having the *current possessor bias*. Those who answered that the toy must be returned to the first possessor in Question (3) in all four scenarios were classified as having the *loan bias*. The percentages of subjects who met the criterion are shown in **Figure 2**.

As shown in **Figure 2**, for both young children and adults, only a small proportion of subjects showed the current possessor bias in this study, and there was no significant difference across four age groups, χ^2^(3) = 3.42, *p* > 0.05. In contrast, approximately one-third of the 3- and 4-year-olds showed the first possessor bias, and the proportion was significantly larger than the proportions in 5-year-olds and adults group, χ^2^(3) = 14.16, *p* < 0.01. This result suggests that younger preschoolers were more likely to insist on the right of the first possessor in judging ownership. A high proportion of the youngest group also showed the loan bias, but with age, the proportion decreased significantly, χ^2^(3) = 19.84, *p* < 0.01.

### Justifications for Ownership Judgments

Subjects’ justifications in different conditions are listed in **Table 1**.

**Table 1:** Subjects’ patterns of justification and the number of subjects who give these justifications in each age group.

	3-year old	4-year old	5-year old	adults	χ^2^(3)
**Giving**					
Current state of the object (e.g., “It has been given out.”)	5	7	16	25	28.03^∗∗^
The original possessor’s mind (e.g., “He/she likes it.”)	2	0	0	0	6.64
The current possessor’s mind (e.g., “He/she likes it.”)	1	4	2	0	5.33
The legitimacy of the behavior (e.g., You can’t reclaim something given out)	0	6	3	7	7.75
The property of the object (e.g., “It’s a present.”)	6	6	8	2	4.65
Invalid response.	16	9	3	0	30.55^∗∗^
**Stealing**					
Current state of the object (e.g., “Now he/she got it.”)	5	6	3	0	7.27
Original state of the object (e.g., “It’s originally taken here by him/her.”)	12	12	16	20	3.77
The original possessor’s mind (“He/she likes it.”)	1	1	0	1	1.03
The current possessor’s mind (“He/she likes it.”)	1	4	0	0	9.01^∗^
The legitimacy of the behavior (e.g., “it’s not right to take others’ things without permission.”)	2	4	9	13	11.76^∗∗^
Invalid response.	9	5	4	0	12.00^∗∗^
**Losing**					
Current state of the object (e.g., “Now he/she found it.”)	5	6	5	1	4.44
Original state of the object (e.g., “It’s originally his/hers.”)	11	12	15	17	1.76
The original possessor’s mind (e.g., “He/she just forgot it.”)	4	6	5	6	0.39
The current possessor’s mind (e.g., “He/she likes it.”)	1	2	0	0	3.85
The legitimacy of the behavior (e.g., “Things that picked up should be returned.”)	2	0	3	8	10.70^∗^
Invalid response.	7	6	4	2	4.40
**Abandoning**					
Current state of the object (e.g., “Now he/she found it”)	3	4	3	11	8.71^∗^
Original state of the object (e.g., “It’s originally his/hers.”)	14	12	9	0	20.11^∗∗^
The original possessor’s mind (e.g., “He/she did not want it.”)	4	10	15	23	21.24^∗∗^
The current possessor’s mind (e.g., “He/she likes it.”)	2	0	0	0	6.64
The legitimacy of the behavior (e.g., “It is all right to keep things abandoned”)	0	0	1	0	3.02
Invalid response.	7	6	4	0	8.69^∗^

*^∗^p < 0.05, ^∗∗^p < 0.01*.

We found that subjects mentioned the property of the object only in the giving condition and that no subject referred to the original state of the object to justify their answer in this condition. Many youngest children gave invalid responses in all four conditions. A large proportion of 3-year-olds referred to the original state of the object in all the conditions except for giving. With age, more children referred to the current state of the object in the giving and abandoning conditions and referred to the legitimacy of the behavior in the stealing and losing conditions. Furthermore, they were more likely to refer to the original possessor’s psychological state in the abandoning condition.

Because a mature understanding of ownership means a correct judgment of ownership as well as the reasonable justification for it, we dichotomized subjects’ justifications into appropriate and inappropriate responses based on their answers to Question (3). Subjects’ responses were considered appropriate if they answered Question (3) correctly and provided a reasonable justification. For the giving condition, reasonable justifications included the current state of the toy, the legitimacy of the behavior, and the property of the object. For the stealing condition, reasonable justifications included the original state of the toy and the legitimacy of the taking behavior. For the losing condition, reasonable justifications included the original state of the toy, the legitimacy of the keeping behavior, and the original possessor’s mind. For the abandoning condition, reasonable justifications included the current state of the object, the legitimacy of the keeping behavior, and the original possessor’s mind.

All adults showed appropriate responses in the four conditions, except that two of them failed to answer Question (3) in the losing condition. Therefore, only preschoolers’ responses were analyzed, and they are listed in **Table 2**.

**Table 2:** Proportion of children giving appropriate justifications (%).

Groups	Giving	Stealing	Losing	Abandoning	χ^2^(3)
3-year-olds	26.7	50	16.7	6.7	16.50^∗∗^
4-year-olds	50	50	34.4	37.5	2.64
5-year-olds	75	81.2	56.2	50	9.41^∗^
χ^2^(2)	14.48^∗∗^	8.61^∗^	10.53^∗∗^	14.05^∗∗^	

*^∗^p < 0.05, ^∗∗^p < 0.01*.

For youngest children, the proportions of subjects who showed appropriate responses in the four conditions were very low. The proportion of appropriate responses increased significantly with age in all four conditions and varied across conditions. Whereas 3-year-olds and 5-year-olds showed more appropriate responses in the giving and stealing conditions, relatively fewer of them showed appropriate responses in the losing and abandoning conditions. However, this difference did not reach statistical significance in 4-year-olds.

## Discussion

The ability to identify legitimate transfers and illegitimate transfers of ownership is very important for children’s social adaptation. In this study, we investigated Chinese preschoolers’ ownership judgments in four transfer conditions. We found that children’s ability to identify ownership changes generally improved with age overall. Moreover, the developmental trajectories of children’s ownership judgments varied across conditions. Whereas 3-year-olds understood that ownership cannot be transferred in the stealing condition, such understanding was not attained until 4 years old in the giving condition and 5 years old in the losing and abandoning conditions, at which age their performance approached that of adults. Younger preschoolers found it more difficult to reason about ownership in the losing and abandoning conditions than in the giving and stealing conditions. Three-year-olds and 4-year-olds were found to show the first possessor bias, and 3-year-olds also had the loan bias in judging ownership. The youngest children had difficulty in providing appropriate justifications.

Consistent with the results of previous studies (Friedman and Neary, 2008; Blake and Harris, 2009; Kim and Kalish, 2009), we found that it was harder for younger preschoolers to reason about ownership in transfer contexts, and they displayed an obvious first possessor bias in the study. To judge ownership transfer in the four contexts, the original owners’ intent should be an important concern. Though previous research showed that from an early age, children can understand others’ intent based on their behavioral attempt (e.g., Meltzoff, 1995), children at this age may not be able to overcome the first possession bias in guiding their ownership judgments. Compared with the internal mental state, first possession was visually a more salient cue for young preschoolers. As in the case of moral ownership (Piaget, 1932; Cushman et al., 2013), younger preschoolers might rely more on clues that are visually conspicuous, such as outcome and first possession. With age, children will better understand the role of intent in determining ownership and learn to identify contexts in which ownership can or cannot be transferred. The result suggests that younger preschoolers lack an understanding of the causal relationship between intent and ownership transfers and tend to exaggerate the boundary of the first possession principle in ownership judgments.

As hypothesized, we found that young children’s understanding of ownership was not parallel across the four transfer contexts. First, we found that 3-year-olds performed better in the stealing and losing conditions than in the giving and abandoning conditions, and the difference reached significance between ownership judgments in the stealing and abandoning conditions. Thus, it is easier for preschoolers to identify illegitimate means of ownership than to identify legitimate means of ownership. This may relate to parents’ disciplinary behavior toward children, which may aim to decrease antisocial behavior more than punishing children’s failure to act prosocially (Grusec and Goodnow, 1994). This encourages younger children to be more sensitive to the prohibition of illegitimate transfers than to the enforcement of legitimate transfers and to better understand the ownership state for illegitimate transfers.

Second, younger preschoolers found it even harder to reason about ownership in the losing and abandoning contexts than in the giving and stealing contexts. Whereas 5-year-olds performed significantly better than chance in all four contexts, as did adults, 3- and 4-year-olds performed below or at the level of chance in the losing and abandoning contexts. The first possessor bias predicted younger children would judge ownership correctly in the losing and stealing contexts but not correctly in the giving and abandoning contexts. The results showed young children did judge correctly in the stealing context and did not correctly judge the current processor as the owner in the giving and abandoning contexts. However, 3-year-olds and 4-year-olds were found to have difficulty identifying the first-possessor as the owner of a lost object, which seems to be inconsistent with the general first possessor bias the study found. Cram and Ng (1989) found that many young preschoolers tended to support the finder in keeping a found object and that they justified their answers with reference to physical contact (“He/she found it”). This is also the case in our study. Many children who judged the current possessor as the owner in the losing condition justified their responses with “He/she found it.” Thus, ownership judgments of younger preschoolers could be influenced by either physical contact or first possession bias, which may have led to their chance-level performance in the losing context at the group level.

In contrast to previous studies, in addition to the first possession bias, we also found a loan bias in the youngest group, which was not reported in Western samples and might be related to the Chinese culture. Three-year-olds tended to answer that the toys should be returned to the original owner and referred to the original state of the object to justify their answers in nearly all conditions. The Chinese culture discourages people from keeping things that are not acquired with effort (in Chinese, 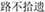 Lu bu shi yi). Just as a children’s popular song goes, “I picked up a penny on the side of the road and handed it over to the police uncle.” Chinese children are exposed to such socialization practices from an early age, which may contribute to the differences in ownership reasoning patterns of Chinese children relative to their counterparts in Western countries.

### Limitations

This study has some limitations. First, we recruited only Chinese subjects and did not include Western subjects in the study. Thus, direct cultural comparisons were not feasible. Future studies should examine cross-cultural differences by including participants from multiple cultures. Second, since we used a cross-sectional study design, we cannot test the true developmental trajectories of children’s ownership judgments. Future studies can adopt a longitudinal design to follow the same group of children over time so that the developmental trajectories of children’s ownership reasoning can be more rigorously tested.

## Conclusion

Despite these limitations, this study has several useful implications. Object disputes are among the most frequent interpersonal conflicts among preschoolers (Ross and Conant, 1992). We found that younger children’s judgments of ownership in different transfer conditions were not as developed as that of older children, which may partly explain the frequent toy conflicts that occur in preschool. Younger children may not be able to identify conditions where ownership has been transferred or not, and may thus wrongly claim ownership status and incur disputes. We found that younger children often used the first possessor bias to reason about ownership in this study. A possibly useful way to decrease children’s object disputes is to instruct them on how to distinguish conditions where the first possessor principle can and cannot be applied. To conclude, we found that Chinese preschoolers’ ability to identify the legitimacy of ownership transfers increased with age, and this ability was dependent on the type of transfer context. Younger Chinese children tend to use the first possessor and loan bias to reason about ownership in transfer conditions.

## Ethics Statement

This study was carried out in accordance with the recommendations of Scientific Research Ethics Committee of Institute of Psychology, CAS. The protocol was approved by the Scientific Research Ethics Committee of Institute of Psychology.

## Author Contributions

ZL and MQ designed the study, collected and analyzed data, and drafted the paper. LZ co-designed and revised the paper critically. JY revised the paper critically.

## Conflict of Interest Statement

The authors declare that the research was conducted in the absence of any commercial or financial relationships that could be construed as a potential conflict of interest.

## Appendix

Scenarios and corresponding pictures used in the study.
